# Enriched G4 forming repeats in the human genome are associated with robust well-coordinated transcription and reduced cancer transcriptome variation

**DOI:** 10.1016/j.jbc.2024.107822

**Published:** 2024-09-26

**Authors:** Ruth B. De-Paula, Albino Bacolla, Aleem Syed, John A. Tainer

**Affiliations:** 1 Graduate School of Biomedical Sciences, Baylor College of Medicine, Houston, Texas, USA; 2 Department of Molecular and Cellular Oncology, The University of Texas MD Anderson Cancer Center, Houston, Texas, USA; 3 Molecular Biophysics and Integrated Bioimaging, Lawrence Berkeley National Laboratory, Berkeley, California, USA

**Keywords:** G-quadruplex, epigenetics, bioinformatics, gene expression, cancer, inherited disease, non-B DNA

## Abstract

Non-B DNA G-quadruplex (G4) structures with guanine (G) runs of 2 to 4 repeats can trigger opposing experimental transcriptional impacts. Here, we used bioinformatic algorithms to comprehensively assess correlations of steady-state RNA transcript levels with all putative G4 sequence (pG4) locations genome-wide in three mammalian genomes and in normal and tumor human tissues. The human pG4-containing gene set displays higher expression levels than the set without pG4, supporting and extending some prior observations. pG4 enrichment at transcription start sites (TSSs) in human, but not chimpanzee and mouse genomes, suggests possible positive selection pressure for pG4 at human TSS, potentially driving genome rewiring and gene expression divergence between human and chimpanzee. Comprehensive bioinformatic analyses revealed lower pG4-containing gene set variability in humans and among different pG4 genes in tumors. As G4 stabilizers are under therapeutic consideration for cancer and pathogens, such distinctions between human normal and tumor G4s along with other species merit attention. Furthermore, in germline and cancer sequences, the most mutagenic pG4 mapped to regions promoting alternative DNA structures. Overall findings establish high pG4 at TSS as a human genome attribute statistically associated with robust well-coordinated transcription and reduced cancer transcriptome variation with implications for biology, model organisms, and medicine.

The discovery of right-handed B-form DNA is a breakthrough biological paradigm, challenging preceding concepts on trait heritability and protein production. Since then knowledge about DNA has shaped how we study genetics, influencing studies of genes and inherited features. Yet, canonical B-form DNA base-paring and stacking architecture in its static form has historically contrasted with dynamic and polymorphic noncanonical DNA folds observed *in vivo*, collectively known as non-B DNA (1, 2), and its association with epigenetic marks (3, 4, 5, 6). In particular, guanine repetitions, which are widespread in all domains of life, may spontaneously form G-quadruplexes (G4) (7).

G4s are non-B DNA or RNA structures composed of four runs of repeated guanines (generally 2–4), each interrupted by up to 22 random bases (8, 9). G4 structures are stabilized by alkali ions such as potassium (K^+^), sodium (Na^+^), and magnesium (Mg^2+^), at physiological concentrations (10, 11, 12). Putative G4-forming sequences (pG4s) comprise 0.3% of the human genome and are enriched (*i.e.* more prevalent in specific regions than in the genome as a whole) at promoters, splice sites and 5′UTRs of genes, where they have been associated with either negative or positive regulation of gene expression (8, 13, 14, 15, 16, 17, 18, 19), control of alternative splicing (20), and negative regulation of topoisomerase I activity (21). G4s have also been related to genome instability, enabling germline mutations leading to genetic disorders, or somatic mutations in cancer (22, 23, 24, 25, 26, 27, 28, 29). In addition, G4 structures have been linked to the pathology of neurological and repeat expansion disorders, such as cerebellar ataxia, neuropathy, vestibular areflexia syndrome (CANVAS), and fragile X syndrome (30, 31). G4s potentially act at replication forks, which must be protected, reversed, and restarted by helicases, ATPases, nucleases, and adaptors if stalled by non-B DNA structures or mutations (32, 33, 34, 35, 36). In particular, analyses of pG4 effects on gene expression (23, 37, 38, 39, 40) have provided a contrasting picture suggesting cases of positive (40, 41) and negative (13, 17, 18, 42) regulation. Therefore, a systematic global analysis of pG4 at different gene locations and their association with gene expression and its variability among different individuals appears warranted.

Here, we applied a comprehensive bioinformatic approach to assess pG4 association with gene expression levels genome-wide. We used a stratified analysis of location-specific effects of pG4 at different gene regulatory loci that control transcription (promoter region and transcription start sites (TSSs)) and translation (5′UTR, 3′UTR, exons and introns). Given the strikingly higher cancer rates in humans compared to chimpanzees, despite their recent evolutionarily divergence (∼7 million years ago) (43), we reasoned that comparison of genomic pG4 in human with chimpanzee and mouse, as model organisms for disease and cancer research, may be informative. We found that pG4s were enriched genome-wide at TSSs of expressed sequence tags (ESTs) comprising both mapped and unmapped transcripts in humans, but not in the chimpanzee and mouse. Furthermore, mapped genes containing pG4 at any locus displayed higher expression levels and lower expression variability in available databases than genes without pG4s. Notably, olfactory/chemical stimulus-related genes were poor in pG4s, whereas neuronal- and developmental-related genes were rich in pG4s. Interestingly, many germline and somatic pathogenic mutations fell within pG4 regions, including super hotspots in cancer, possibly perturbing G4 stability and interactions. Overall, we found robust transcription by pG4-containing genes associated with significantly low variability in gene expression in both health and disease contexts.

## Results

### TSSs of human RNA-coding regions are enriched for pG4

As first steps to understand the dynamics of G4 structures, we scanned the genome of three mammalian species for pG4s with in-house search algorithms. Our basic algorithm, named C++Quad, resembles the widely used Quadparser (8) and the C-based algorithm used in nonB-DB (44, 45), but it was modified to parse for noncanonical repeats. In addition, the code was parallelized (message passing interface (MPI); https://www.mcs.anl.gov/research/projects/mpi/) for faster retrieval of the data.

Between ∼10 and 16 million pG4s sequences were found in the human (hg38 assembly), chimpanzee (panTro6 assembly), and mouse (mm10 assembly) genomes, attesting to the widespread distribution of such elements (Table 1). The mouse genome was two times richer in pG4 sequences (0.62% of total genomic base pairs) than the human and chimpanzee genomes (0.34% and 0.33%, respectively), supporting previous work (44, 46). Since G4s are regarded as important gene expression modulators, we next screened these three genomes for pG4s overlapping TSSs of ESTs comprising transcripts for both annotated and nonannotated genes.

**Table 1:** TSSs of human ESTs are enriched for pG4

pG4 statistics – Human	
Number of pG4 bp in hg38	10,002,935
Total number of bp in hg38^a^	2,948,583,725
Number of ESTs with start site at pG4 DNA	54,397
Total number of ESTs in hg38^b^	8,624,218
Percentage of hg38 with pG4 (%)	0.34
Percentage of hg38 without pG4 (%)	99.66
Percentage of ESTs with start site in pG4 (%)^b^	0.63
Percentage of ESTs without start site in pG4 (%)^b^	99.37
Fisher test significance	*p* < 0.0001
pG4 statistics—Chimpanzee	
Number of pG4 bp in panTro6	9,874,095
Total number of bp in panTro6^a^	3,018,592,990
Number of ESTs with start site at pG4 DNA	30
Total number of ESTs in panTro6^b^	17,848
Percentage of panTro6 with pG4 (%)	0.33
Percentage of panTro6 without pG4 (%)	99.67
Percentage of ESTs with start site in pG4 (%)^b^	0.17
Percentage of ESTs without start site in pG4 (%)^b^	99.83
Fisher test significance	*p* < 0.0001
pG4 statistics—Mouse	
Number of pG4 bp in mm10	16,373,835
Total number of bp in mm10^a^	2,652,767,259
Number of ESTs with start site at pG4 DNA	10,786
Total number of ESTs in mm10^b^	4,341,907
Percentage of mm10 with pG4 (%)	0.62
Percentage of mm10 without pG4 (%)	99.38
Percentage of ESTs with start site in pG4 (%)^b^	0.25
Percentage of ESTs without start site in pG4 (%)^b^	99.75
Fisher test significance	*p* < 0.0001

a Ungapped length.

b Without considering chrM and unmapped chromosomes.

The human EST library was the most well represented, with >8 million entries, compared to mouse (>4 million entries) and chimpanzee (∼18,000 entries). Our analysis revealed a greater percentage of ESTs with pG4s overlapping TSS in humans (0.63%), compared to mouse (0.25%) and chimpanzee (0.17%). Given the different abundance of pG4 bases in these three species, the “enrichment” of pG4 at TSS in humans would translate into a factor of 1.85 (0.63%/0.34%), compared to 0.40 (0.25%/0.62%) in the mouse and 0.52 (0.17%/0.33%) in the chimpanzee. With respect to the small library size in chimpanzee, we confirmed that pG4 enrichment at human EST TSS was independent on library size (Table S1). These combined analyses support the view that G4-forming sequences may have been under positive selection pressure at TSSs along the human lineage.

### Genes with pG4 display robust expression and are enriched in neuronal and developmental genes

**Figure 1:**
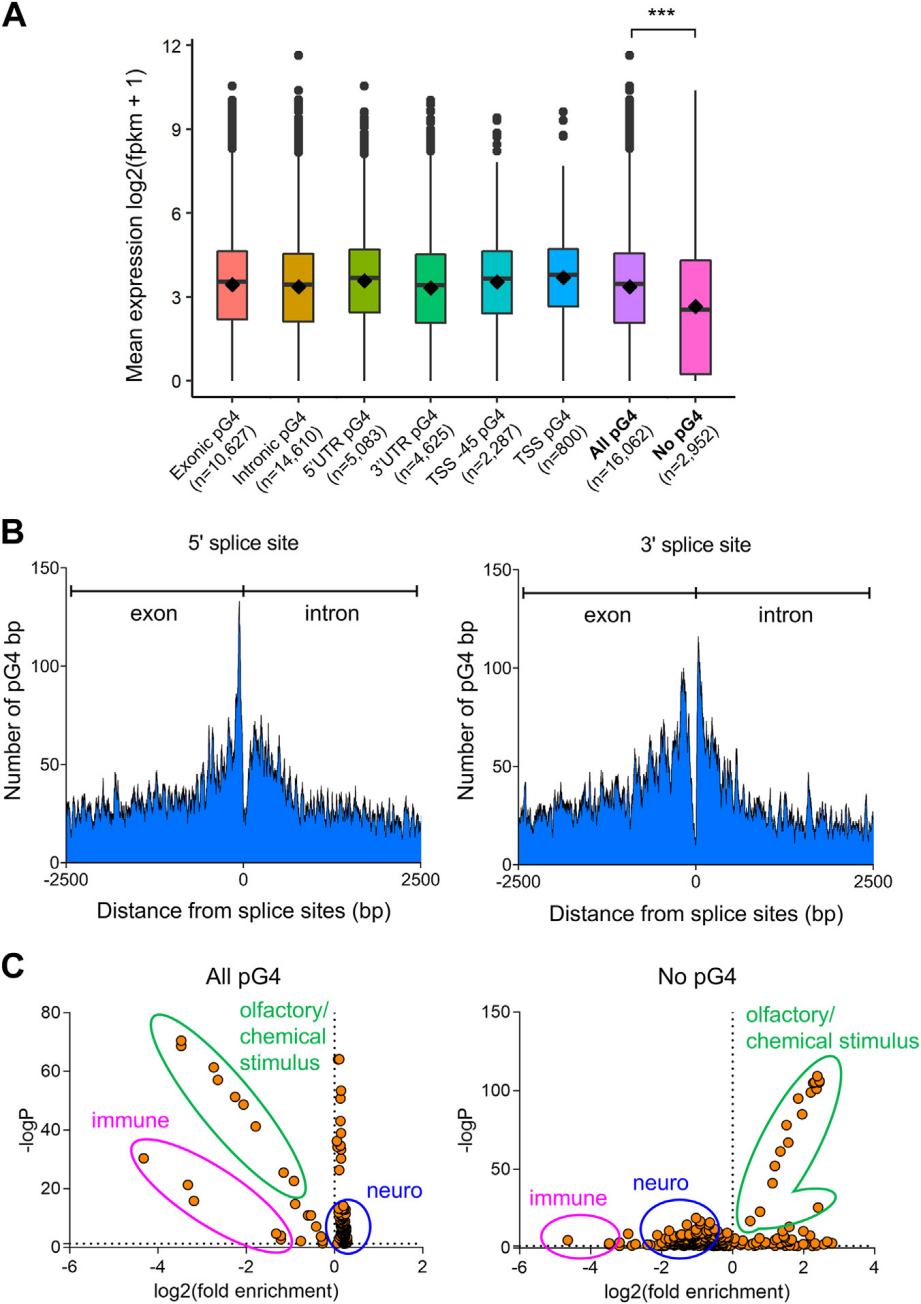
**Genes with pG4 display robust expression, are depleted in olfactory/chemical stimulus-related genes and are enriched in neuronal- and developmental-related genes.***A*, boxplots representing the expression levels of genes with and without pG4s at different regulatory locations. Expression levels were converted to log2 (fpkm + 1) and results from different samples were averaged. Boxplots were constructed using the interquartile range (IQR) and median, and whiskers were calculated using IQR ∗ 1.5. All comparisons between genes with and without pG4 were statistically significant according to Wilcoxon tests. ∗∗∗, Wilcoxon test *p* < 0.001. *B*, Cartesian plots representing the genome-wide counts of pG4 bp within 2500 bp before and 2500 bp after 5' (*left*) and 3' (*right*) splice sites. *C*, scatter plots displaying GO terms scattered according to log2 of fold enrichment (*x* axis) and -log10 of *p*-values (*y* axis). *Circled in green*, olfactory/chemical stimulus-related terms; *circled in magenta*, immune-related terms; *circled in blue*, neuronal- and developmental-related terms. GO, Gene Ontology.

To test for genome-wide and location-specific effects of putative G4 structures on gene expression, we mapped coordinates of the main gene regulatory regions (exons, introns, 5′UTR, 3′UTR, TSS-45, and TSS) and intersected them with the C++Quad pG4 coordinates. This approach enabled us to perform a gene expression evaluation for genes containing pG4 at different locations in normal human tissues using The Human Protein Atlas (47). For each gene analyzed, we have calculated the mean of the expression across all evaluated tissues. Our results revealed that genes with pG4s at exons, introns, 5′UTR, 3′UTR, TSS-45, and TSS were expressed at higher levels than at the corresponding locations of genes without pG4 (Fig. 1*A*; Table S1). About 77% of human annotated genes contained pG4s in introns (14,610 out of 19,014 genes). To assess whether this finding was simply due to the larger size of introns compared to exons and UTRs or reflected a biological role, we performed a genome-wide screen to determine the prevalence of pG4s around splice sites. Interestingly, the analysis revealed a progressive enrichment of pG4s as intron/exon boundaries were approached, both from the 5′ as well as the 3′ sites, as previously reported (20), although splice junctions themselves were excluded from pG4 motifs (Fig. 1*B*). pG4 enrichment reached a maximum of ∼4-fold at 56 bp before 5′ splice sites and 34 bp after 3′ splice sites, when pG4 bases at splice junctions were compared to the average number of pG4 bp in the ±2500 bp flanking areas of the splice sites.

Having assessed the impact of pG4 on gene expression levels in the normal population, we then grouped genes with and without pG4 according to their biological function by using Gene Set Enrichment Analysis (GSEA). While supporting and extending previous reports that genes with pG4s are linked to transcription factor activity, development, and cell-cell signaling (48, 49, 50), our findings revealed that genes with pG4 were furthermore enriched for a wider range of Gene Ontology (GO) terms (Table S2). When considering the strongest enrichment and depletion ratios, genes with pG4 were depleted in both immune- and olfactory/chemical stimulus-related terms, while genes without pG4 were enriched in olfactory/chemical stimulus-related GO terms (Fig. 1*C*). Neuronal- and developmental-related terms were most enriched in pG4-containg genes (∼1.2). Thus, pG4 distribution within gene families was nonrandom, being most enriched in neurogenesis- and brain development-related genes and most depleted in olfactory receptor (OR) genes. Together these data imply that pG4s may represent functional genetic elements for the control of transcription and splicing.

### pG4-containing genes display low expression variability among individuals and genes

**Figure 2:**
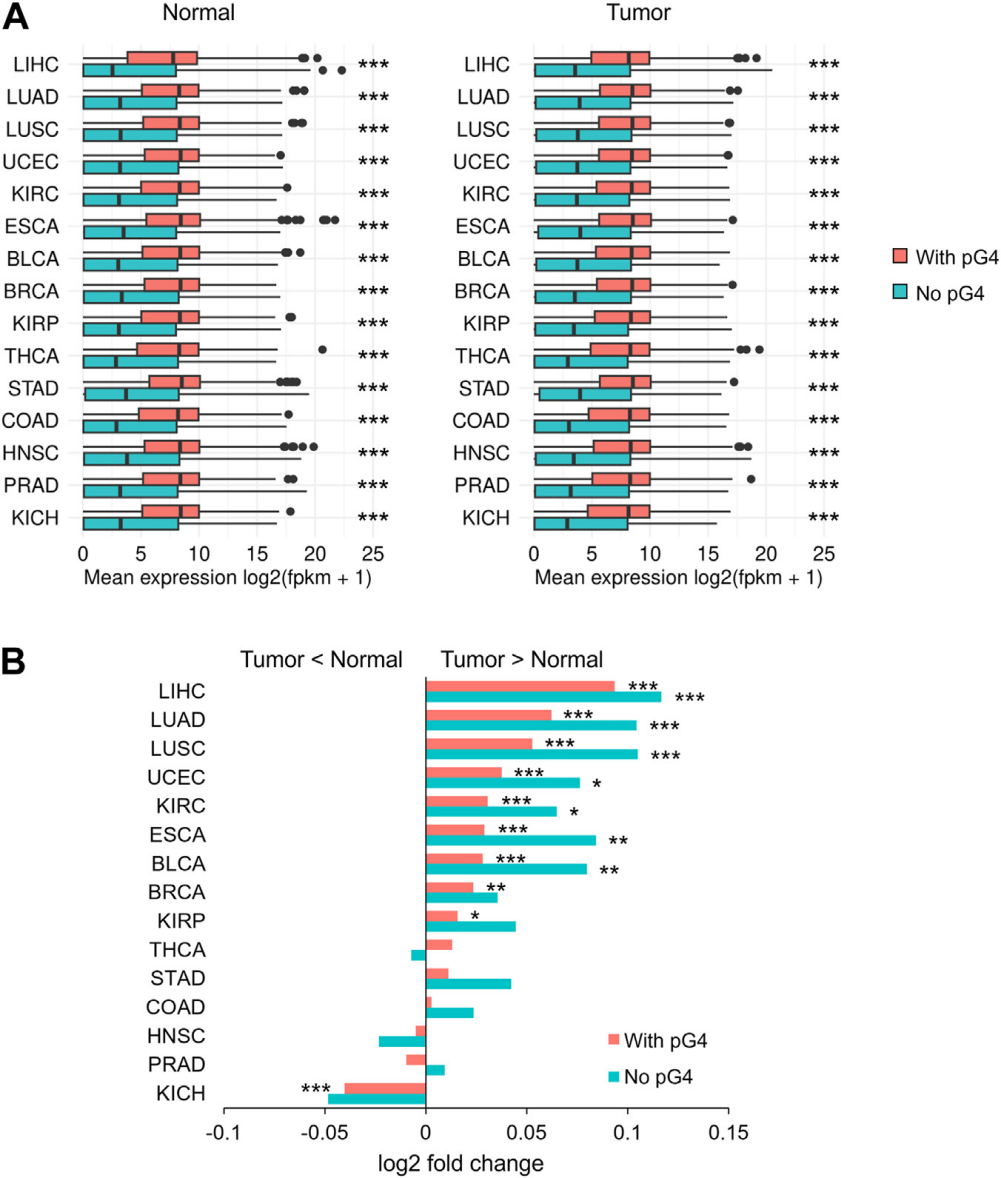
**Genes with pG4 have lower expression difference between tumors and normal tissue.***A*, box plots representing the expression levels of genes with and without pG4s in normal (*left*) and tumor (*right*) tissue. Expression levels were converted to log2(fpkm + 1) and results from different samples were averaged. Boxplots were constructed using the interquartile range (IQR) and median, and whiskers were calculated using IQR ∗ 1.5. All comparisons between genes with and without pG4 were statistically significant according to Wilcoxon tests. ∗∗∗, Wilcoxon test *p* < 0.001. *B*, columns graph representing the log2 fold change difference of expression among tumor tissues and normal control tissues in TCGA. Bars on the *left* part of the graph represent cases in which the average expression of genes was higher for normal than for tumor tissue, and bars on the *right* part represent the opposite. ∗, *t* test *p* < 0.05; ∗∗, *t* test *p* < 0.01; ∗∗∗, *t* test *p* < 0.001. BRCA, breast carcinoma; BLCA, bladder urothelial carcinoma; COAD, colon adenocarcinoma; ESCA, esophageal carcinoma; HNSC, head and neck squamous cell carcinoma; KIRC: kidney renal clear cell carcinoma; KIRP, kidney renal papillary cell carcinoma; KICH, kidney chromophobe carcinoma; LIHC, liver hepatocellular carcinoma; LUAD, lung adenocarcinoma; LUSC, lung squamous cell carcinoma; PRAD, prostate adenocarcinoma; STAD, stomach adenocarcinoma; TCGA, The Cancer Genome Atlas; THCA, thyroid carcinoma; UCEC, uterine corpus endometrial carcinoma.

As tumorigenesis entails a profound reprogramming of gene expression profiling and we found that pG4s are associated with enhanced transcription, we examined whether predicted G4 structures may impact gene expression changes in cancer. To this end, we compared global transcriptomic profiles from RNA-seq data for genes with and without pG4s between tumor and matched controls from The Cancer Genome Atlas (TCGA) repository (Fig. 2*A*; Table S1). Mirroring what was observed in healthy tissues, the global pattern of expression was consistently higher for pG4-containing genes than for genes without pG4, both in matched controls and in the tumor tissues. The average gene expression was higher in most tumors than in matched controls, but the tumor-to-normal fold change was smaller for genes with pG4 than for those without pG4 (Fig. 2*B*; Table S1). Even for kidney chromophobe, which displayed an unusual shift in transcription profiling characterized by lower expression in the tumor than in matched controls, genes with pG4 underwent a milder change in expression than genes without pG4.

To further elucidate the correlation between gene expression and pG4 motifs, we assessed the variability in gene expression by computing the coefficient of variation (CV), *i.e.* [(standard deviation (SD) of expression/mean expression) ∗100] for each gene across individual TCGA patients (indiv-CV), both in the tumor and in matched controls. The indiv-CV was consistently smaller for pG4-than for non pG4-containing genes, both in tumors and in matched controls (Fig. S1). We interpret this finding to indicate that in both disease and healthy states the expression of pG4-containing genes varies within tighter boundaries than for non pG4-containing genes. However, indiv-CV values were usually greater in tumors than in matched controls, both for pG4-and non-pG4-containing genes, likely reflecting a pervasive dysregulation of gene expression in tumorigenesis (Fig. S2).

**Figure 3:**
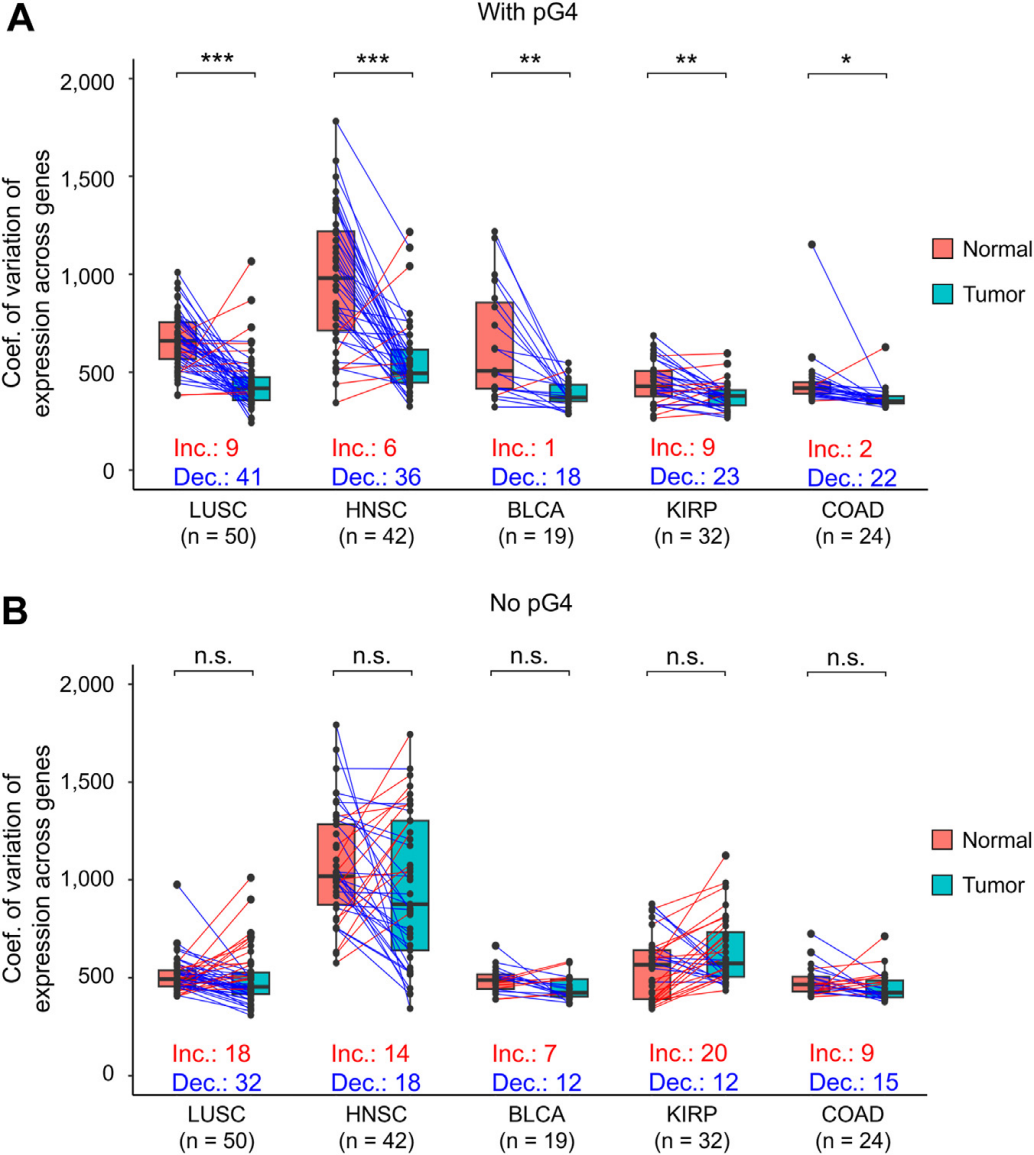
**Expression variability of pG4-containing genes decreases in tumors relative to matched controls.***A*, box plots for selected cancers representing coefficients of variation of expression across pG4 genes (g-CV). Coefficients were calculated per individual sample, and only samples with both tumor and normal tissue data were analyzed. Box plots were constructed using the interquartile range (IQR) and median, and whiskers were calculated using IQR ∗ 1.5. Paired *t* tests were used to compare the coefficients between normal and tumor tissue. ∗, *t* test *p* < 0.05; ∗∗, *t* test *p* < 0.01; ∗∗∗, *t* test *p* < 0.001. Matched samples are connected by *lines*. *Red lines*: increased variability in tumor compared to normal tissue; *blue lines*: decreased variability in tumor compared to normal tissue. *B*, boxplots for the same cancers as in panel A, representing coefficients of variation of expression across genes without pG4. CV, coefficient of variation.

To further test these findings, for each patient we computed the CV across genes (g-CV), for both genes with and without pG4. Then we compared the data between normal and tumor samples for all available cancer types (Table S1) for which the number of normal-tumor pair samples was at least 15. In five types of cancer, g-CV decreased significantly in the tumor compared to the matched control for pG4-containing genes (Fig. 3*A*), whereas no differences were observed for non pG4-containing genes (Fig. 3*B*). These composite data indicate that pG4 motifs are likely associated with a tighter control of gene transcription genome-wide than genes without such motifs. Therefore, on one hand, association with pG4 motifs is seen to lead to an increase in gene expression genome-wide; on the other hand, it reduces gene expression level variation during the process of tumorigenesis. This reduction is observed both in terms of interindividual variability and in terms of expression range for the transcriptome within an individual sample in selected tumors.

### Disease-related mutations target unstable germline pG4 but stable cancer genome pG4 structures

**Figure 4:**
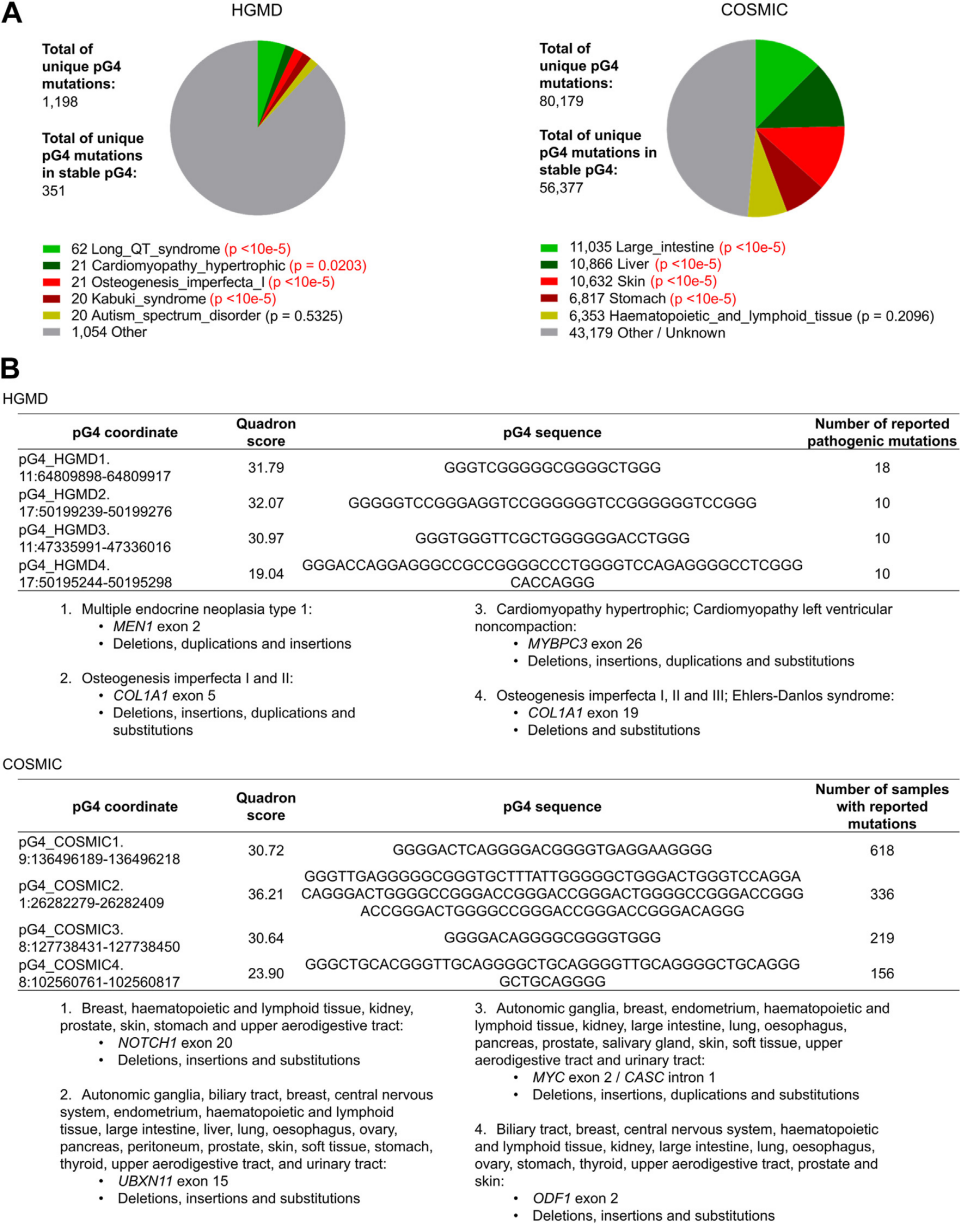
**Diseases with cardiac symptoms and cancers of the gastrointestinal and aerodigestive systems are enriched for mutations overlapping pG4 motifs.***A*, most common inherited diseases associated with pG4 mutations identified by C++Quad search algorithm. For COSMIC search, only the primary tumor site was considered. *B*, most common and stable pG4s identified by Quadron and their associated mutations. The number of mutations for HGMD is based on unique genomic mutation coordinates, and for COSMIC is based on unique combination of genomic mutation coordinates and sample name. HGMD, Human Gene Mutation Database.

The formation of non-B DNA structures, including G4s, can promote processes that induce single nucleotide polymorphism (SNP) (51, 52) and architectural polymorphisms, such as translocations and deletions, which are often associated with genetic disease (28, 53, 54, 55, 56). Yet, sequence-based pG4 search algorithms such as ours raise the question as to whether such motifs do fold into G4 structures of varying stability in cells. Therefore, we used Quadron (Q) (https://quadron.atgcdynamics.org), a bioinformatics tool that allows for quantitative assessment of experimentally based stability scores, to associate G4 stability scores to pG4s that intersected mutations from (1) Human Gene Mutation Database (HGMD) for germline mutations linked to inherited diseases and (2) Catalogue of Somatic Mutations in Cancer (COSMIC) (Fig. 4; Table S3) for somatic mutations accumulated in tumors.

The HGMD database revealed 1198 mutations overlapping pG4 regions (∼1.5 fold-enrichment when compared to genome average), several of which were associated with pathological conditions (Fig. 4*A*). Genetic diseases most associated with pG4 mutations were long QT syndrome (*p* < 1 × 10e-5), cardiomyopathy hypertrophic (*p* = 0.0203), osteogenesis imperfecta (*p* < 1 × 10e-5) and the Kabuki syndrome (*p* < 1 × 10e-5). Although many pG4 mutations were linked to autism spectrum disorder, there was no significant enrichment of those mutations compared to genome-wide average (Fisher test *p* = 0.5325) (Table S1). However, of the 1198 pG4 mutations, only 351 (29.21%) were located at pG4 sequences predicted to form stable G4 structures (s-pG4, Q-score ≥19), which is less than expected when considering that 51.36% of all pG4s genome-wide are classified as stable using a Q-score ≥19.

Next, we selected some s-pG4 sequences rich in mutations (*i.e.* pG4 mutation hotspots) to investigate their biological context. The pG4_HGMD1 located at *MEN1* exon 2 contained 18 germline mutations linked to susceptibility to multiple endocrine neoplasia type 1. These mutations were classified as deletions, duplications, and insertions. pG4_HGMD2, pG4_HGMD3, and pG4_HGMD4 comprised ten mutations each. pG4_HGMD2 and pG4_HGMD4 are spaced by around 4000 bp in *COL1A1*, the first being located at exon 5 and the second at exon 1. *COL1A1* is linked to bone formation disorders (osteogenesis imperfecta and Ehlers-Danlos syndrome). These bone disorders are also associated with cardiovascular symptoms, such as poor heart valve function and increased heart rate after standing up, characteristics which were also shared by pG4_HGMD3-linked disorders (cardiomyopathy hypertrophic and cardiomyopathy left ventricular noncompaction) (57, 58, 59, 60).

We furthermore tested whether pG4 mutations linked to long QT syndrome, osteogenesis imperfecta, and Kabuki syndrome may alter G4 folding dynamics, by inspecting 36 substitutions associated with those diseases and their potential to perturb secondary structure formation. Of the 36 mutated pG4s examined, 31 (86.1%) had alteration of at least one of the three metrics examined (minimum ΔG, maximum melting temperature, and number of secondary structures formed). A total of 14/36 substitutions (38.9%) led to a decrease in G4 structure stability, as predicted from a weaker ΔG, 15 (41.7%) led to a decrease in G4 melting temperature, and 15 (41.7%) led to a decrease in the number of secondary structures potentially formed (Tables 2 and S3).

**Table 2:** Substitutions in pG4s within multiple cardiac-related genes alter secondary structures stability

pG4 coordinate + 20 bp flank (hg38)	pG4 strand	Gene	Disease	Substitution coordinate (hg38)	Min. ΔG	Max. melting	N of secondary structures
chr11:2445235-2445338	−	*KCNQ1*	Long QT syndrome	chr11:2445268G>T	↓	↓	↓
				chr11:2445270G>C	↓	↓	↓
				chr11:2445295C>T	↓	=	↓
				chr11:2445315C>A	=	=	=
chr3:38550463-38550522	+	*SCN5A*	Long QT syndrome	chr3:38550499C>T	=	=	=
				chr3:38550500G>A	=	=	↑
chr3:38555638-38555702	+	*SCN5A*	Long QT syndrome	chr3:38555682G>T	↑	↓	↑
				chr3:38555682G>A	↑	↓	=
chr7:150946880-150946951	+	*KCNH2*	Long QT syndrome	chr7:150946905G>A	↑	↑	↑
				chr7:150946929G>A	↓	↑	=
chr7:150947400-150947464	+	*KCNH2*	Long QT syndrome	chr7:150947435G>T	=	=	↓
				chr7:150947440G>A	=	=	↓
chr7:150947766-150947832	−	*KCNH2*	Long QT syndrome	chr7:150947790C>T	↑	↓	↑
				chr7:150947791C>T	↑	↓	↓
				chr7:150947798C>T	↑	↓	↑
				chr7:150947800C>T	↓	↓	↓
				chr7:150947800C>G	↓	↓	↑
				chr7:150947801C>A	=	↓	=
				chr7:150947806C>T	↑	↓	↓
				chr7:150947807G>A	↑	↓	=
				chr7:150947812C>T	↓	=	↑
chr7:150947803-150947867	−	*KCNH2*	Long QT syndrome	chr7:150947833G>A	↑	↓	↓
chr7:150950129-150950189	−	*KCNH2*	Long QT syndrome	chr7:150950168C>T	=	=	↓
				chr7:150950168C>G	=	=	↑
				chr7:150950168C>A	=	=	=
chr1:42746734-42746800	+	*P3H1*	Osteogenesis imperfecta	chr1:42746765G>A	↑	↓	↑
chr17:50199219-50199296	+	*COL1A1*	Osteogenesis imperfecta	chr17:50199255C>A	↑	↓	=
chr12:49,027,043-49027110	+	*KMT2D*	Kabuki syndrome	chr12:49027070G>A	↓	↑	↓
				chr12:49027073C>T	=	=	↓
				chr12:49027084G>T	↓	↑	↓
				chr12:49027088G>A	↑	↑	↓
chr12:49031667-49031751	+	*KMT2D*	Kabuki syndrome	chr12:49031704G>A	↓	↑	=
chr12:49038517-49038586	+	*KMT2D*	Kabuki syndrome	chr12:49038543G>A	=	=	=
chr12:49042262-49042329	+	*KMT2D*	Kabuki syndrome	chr12:49042305C>A	=	=	=
chr12:49053021-49053085	−	*KMT2D*	Kabuki syndrome	chr12:49053047C>A	↑	↑	↓
				chr12:49053059C>T	↑	↑	=

A similar search performed in the COSMIC dataset revealed that of the 80,179 unique cancer-related pG4 mutations (∼1.92 fold-enrichment when compared to genome average), 56,377 (70.3%) were classified as s-pG4 (Fig. 4*A*). Large intestine (11,035 mutations; 16.0%; Fisher *p* < 10e-5), skin (10,632 mutations; 15.3%; Fisher *p* < 10e-5), and stomach (6817 mutations; 9.3%; Fisher *p* < 10e-5) displayed a greater frequency of pG4 mutations than genome average, whereas in hematopoietic and lymphoid tissues the frequency was not different than expected by chance (6353 mutations; 8.6% observed *versus* 8.5% expected; Fisher *p* = 0.2096). Although tumors of the liver were the second cancer type with most pG4-overlapping mutations (10,866) the percent of mutated pG4 sequences was lower than expected (15.7% observed *versus* 18.0% expected; Fisher *p* < 10e-5).

The s-pG4 with highest mutability was pG4_COSMIC1 in exon 20 of the *NOTCH1* gene, with 618 mutations recurring in different individuals (Fig. 4*B*). Those mutations represent a variety of malignant tissues, including breast, hematopoietic and lymphoid tissue, kidney, prostate, skin, stomach, and upper aerodigestive tract. Other s-pG4s with high number of mutations were pG4_COSMIC2 (336 mutations at *UBXN11* exon 15), pG4_COSMIC3 (219 mutations at *MYC* exon 2 and *CASC* intron 1), and pG4_COSMIC4 (156 mutations at *ODF1* exon 2): all linked to a wide range of malignancies.

Overall, our analysis pointed to a distinct difference between germline and somatic disease mutations. Germline mutations leading to inherited disease tended to exclude pG4 sequences and generally occurred at tracts that are not expected to form stable G4 structures. However, germline mutations associated with pG4 may often strengthen the stability of the ensuing G4 structures or the ability of the pG4 motif to fold into competing non-B DNA secondary structures. By contrast, somatic mutations in cancer displayed tumor-type preferences for pG4 motifs, but generally targeted pG4 tracts predicted to form stable G4 structures.

### The most mutable pG4 motifs are embedded in an unstable DNA sequence context

**Figure 5:**
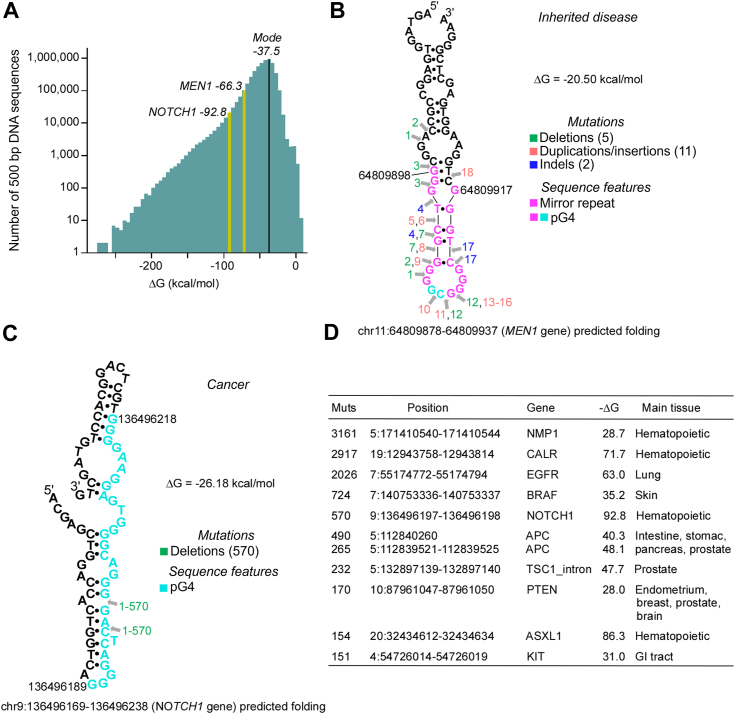
**The most mutable pG4 motifs are embedded in an unstable DNA sequence context.***A*, bar plot of the number of 500 bp bins throughout the entire human genome, sorted by ΔG values. *B*, predicted hairpin structure of the most prominent pG4 hotspot for HGMD mutations, located at *MEN1* gene. Numbers indicate the mutation IDs, and colors indicate types of mutations or sequence features. *C*, predicted hairpin structure of the most prominent pG4 hotspot for COSMIC mutations, located at *NOTCH1* gene. Numbers indicate the mutation IDs, and colors indicate types of mutations or sequence features. *D*, table representing main mutational hotspots (small deletions/insertions/duplications) in cancer. HGMD, Human Gene Mutation Database.

Notably, the frequency of SNPs is not uniform across the human genome, such that the stronger the propensity of DNA motifs within 500 bp to fold into metastable hairpin-loop structures, the higher the number of SNPs in the general population (61). We determined that a histogram of the distribution of the free energy of hairpin-loop formation within contiguous 500 bp bins genome-wide peaked at a ΔG mode of ∼ -37.5 kcal/mol (mean = −20.8 ± 27.5; median −43.5), with a sharp decrease on either side of the distribution (Fig. 5*A*).

To assess if metastable hairpin-loop structures could be associated with pG4 sequences that represent the top hotspots of mutations in the germline (pG4_HGMD1 in exon 2 of *MEN1*) or in cancer (pG4_COSMIC1 in exon 20 of *NOTCH1*), we first mapped on the ΔG histogram the 500-bp bins in which these pG4 sequences were embedded. In both cases, the ΔG was lower than the average values (mode, mean, and median) supporting the notion that the sequence context in which the two pG4 hotspots are located is more unstable than genome-wide average (Fig. 5*A*). Next, we extracted 20 bp of DNA sequence flanking the pG4_HGMD1 and pG4_COSMIC1 sequence motifs and modeled the ensuing DNA into predicted hairpin-loop structures. In both cases, the pG4 tracts were part of a hairpin-loop fold with a rather extensive network of Watson-Crick-type hydrogen bonds and a negative ΔG value, −20.50 kcal/mol in *MEN1* (Fig. 5*B*) and −26.18 kcal/mol in *NOTCH1* (Fig. 5*C*). In addition, the pG4 in *MEN1* was almost entirely formed by two mirror repeats that participated in hairpin-loop folding.

Concerning the distribution of mutations in *MEN1* pG4, apart from mutations 17 and 18, all other variants occurred on the left side of the predicted hairpin stem (Fig. 5*B*), without obvious hotspots. By contrast, in *NOTCH1* pG4, 570 out of 618 variants consisted of a 2-bp deletion at a single site across from a hairpin bulge (Fig. 5*C*), and essentially all variants were discovered from targeted sequencing screens in patients with lymphoma. We further addressed this point of recurrent mutations in cancer by conducting a global survey of recurrent mutational sites in the COSMIC dataset, which revealed that the site in *NOTCH1* pG4 ranked fifth, marking it as a likely prominent mutational hotspot in cancer. Most recurrent mutations were revealed through targeted screens and were most frequently found in hematopoietic malignancies (Fig. 5*D*). Interestingly, of all genomic sites that incurred >150 recurrent mutations in COSMIC, the *NOTCH1* hotspot was the one located in the most unstable 500-bp DNA sequence context. A survey of the COSMIC chromosomal breakpoint dataset revealed that the *NOTCH1* hotspot is a site of recurrent translocation in cancer (chr1:26,282,333, structural ID: COST195256). Thus, we found that the most mutagenic pG4 sequences in the germline and in cancer map into a DNA sequence context that promotes alternative DNA structures, likely allowing for the chromatin environment to synergize with other factors in eliciting recurrent DNA changes in different individuals.

## Discussion

G-quadruplex regions are transient, dynamic, and both cell and tissue-specific (62); however, they can fundamentally impact cell biology outcomes, as seen by G4-associated human diseases and aging processes (63, 64). Thus, G4 structures require detection at replication forks by timeless and unwinding by helicases to maintain genetic and epigenetic stability (65, 66, 67, 68). Resolution of replication fork associated G4s can be mediated by RPA and FANCJ helicase, such that FANCJ deficiency leads to G4 accumulation (69, 70). Still, unresolved G4 loops can be formed cotranscriptionally with G4 DNA on one strand and RNA/DNA hybrids on the other strand (71). Such regions are then subject to oxidative stress and epigenetic marking by G4s (72, 73, 74), indirectly creating mutations and potentially affecting transcription. Importantly, G4s may dysregulate gene expression and have opposing transcriptional impacts (31). We therefore asked whether the overall location of pG4s in three mammalian species and in normal and tumor human tissues may provide general insights on transcriptional impact of pG4 in genomes.

In cell culture, folded G4 structures, as revealed by specific antibody binding and high-resolution genome mapping following Tn5 tagmentation, map preferentially at active promoter and enhancers, both in mouse embryonic stem cells and neural progenitor cells (75). EST libraries aid phylogenetic analyses (76). Here, we found a higher fraction of pG4 sequences overlaps with TSSs in human ESTs than in mouse or chimpanzee ESTs, thereby mirroring the number of promoters harboring pG4 motifs (50). Although a quantitation of folded G4 structures in different cell types in culture shows variability (74), these combined data support the view that the number of G4 structures at TSSs varies among species, and is likely higher in humans than in the chimpanzee or mouse. The extent and mechanisms by which such an enrichment may have contributed to human speciation is a key question raised from our study that we hope may now be tested experimentally worldwide.

Like tandem repeats associated with centromeres (77, 78) and other microsatellite families (79), the population frequency of single nucleotide variants at pG4 is higher than genome-wide average (40, 56), a finding that appears at odds with the persistence of hundreds of thousands of such sequences in extant genomes. As with other repeats, the pool of pG4 motifs in genomes is likely maintained by a balance between decay of existing pG4 motifs and birth of *de novo* pG4 tracts from a library of near-pG4 sequences through common mechanisms (77), such as faulty repair of DNA damage, replication slippage (80, 81), recombination (79, 82), and transposition from G4-containing transposable elements, such as L1 and SINE-VNTR-Alu (27, 83, 84, 85). Damage and mutations at folded G4 motifs likely include those occurring on the complementary strand, where i-motifs may form (86, 87).

Notably, "the microsatellite loci that persist over very long periods are more often found in coding sequences and in regulatory regions” (80). Also, a G4 regulatory role at promoters is supported by their dynamic association with transcription factors at preinitiation complexes (50, 74) and by mutations at pG4 motifs changing gene expression patterns (55). Indeed, folded G4 structures colocalize with transcription regulatory elements enriched with Pol II and open chromatin marks, such as H3K4me2 and H3K4me3 (40, 74). Unstable repeat tracts have been linked to evolutionary tuning of gene expression patterns (88). Thus, given that the strongest GSEA enrichment of functions among human genes containing tandem repeats is regulation of transcription from RNA polymerase II promoters (81), we propose that pG4 may have been playing a more widespread role in gene expression regulation in humans than in the chimpanzee or mouse genomes, a concept now open for future experimental testing.

Experimental analyses in cell culture of pG4 motifs by *in silico*, chromatin immunoprecipitation-sequencing, and massively parallel reporter assays have provided contrasting results. These include both repression and stimulation of transcription (38, 41) plus a role for pG4 in transcriptional pausing (13) and a block of RNA Pol II by G4 structures *in vitro* (31). Alternatively, mapping of G4 structures in cell culture using G4-CUT&Tag technology, which requires more native-like conditions than chromatin immunoprecipitation-sequencing, suggests that although G4 ligand-binding stabilization does restrain nascent RNA transcription, native G4 dynamics segregates with elements linked to active transcription (74, 75). Likewise, chromosomally integrated synthetic reporter genes display higher transcriptional activity when the promoter is coupled to a G4 structure (89).

As transcription factors can form phase-separated condensates that increase the local concentration of reactants (90) and G4 DNA and RNA structures actively participate in phase condensates (91, 92, 93, 94), G4-associated phase separation may contribute to enhancing transcription. By comparing steady-state levels of RNA transcripts in native human tissues and in human tumor tissues, our results support an activating role for pG4 sequences in transcriptional control genome-wide. Our transcriptome profiling included 9326 tumor samples and 713 matched controls comprising 33 types of tumors and 15 matched control tissues from TCGA, and 124 samples from normal individuals of the Swedish population representing 33 organs. Resulting TCGA analyses revealed that genes harboring pG4 sequences are expressed at higher levels than genes without such motifs in both tumor and healthy cells. Thus, although G4 structures may pause or block RNA Pol II progression (13) and repress oncogene (such as *MYC* and *KRAS*) transcription (17, 18), our analyses concur with G4 mapping experiments and *in silico* analyses (19, 40, 95, 96) by showing that G4 structures are linked to enhanced gene expression genome wide and in multiple tissues. For example, pG4 sequences from glutathione peroxidase 4 (*GPX4*) and chloride intracellular channel 4 (*CLIC4*) genes were inserted into the chromatin of Flp-In-293 human cell line before an eGFP reporter gene promoter. These experimental results showed that the pG4s adopted G4 structures and were associated with increased transcription for these few genes consistent with our genome-wide computational association (89). However, experimental validation as to whether G4 structures enhance transcription on a genome-wide scale is challenging. On average, the median for pG4-containing genes is ∼1.5-fold higher than for non-pG4-containing genes, which is statistically valid for the very large genome-wide dataset and furthermore may have quantitative biological effects. However, experimental tests of this difference will require many replicates and a large representative set of genes.

Near splice sites, as well as at UTRs, pG4s on the coding strands retain their ability to fold into G4 structures on the ensuing pre-mRNAs (*i.e.* RNA-G4 (rG4)), where their characteristic high stabilities may be propagated. rG4s near splice sites may indirectly contribute to gene expression by allowing transcription initiation from noncontiguous regions (62, 97). Indeed, our data shows mutual relationships between pG4s, increased expression, and splice site neighboring regions, supporting and extending previous work (20, 37, 40). Factors such as oxidative stress, thermal shock, and starvation may shift rG4 concentration to high levels and lengthen the half-live of mRNA molecules with rG4 in 3′UTRs (98). Thus, rG4 in mRNA may contribute to enhancing gene expression by increasing transcription initiation from noncontiguous regions and by extending mRNA stability.

Regions with folded G4 structures and low methylation rates coincide, supporting a positive regulatory role of pG4s in gene expression (75, 99). Intriguingly, both in blood and skin, methylation rates scale strongly with the maximum lifespan in diverse mammalian lineages (100), raising the possibility that pG4 are part of epigenetic mechanisms linked to shared evolutionary constraints on lifespan. Therefore, the dynamics of G4 formation is a potential underappreciated pivotal point in the regulation of gene expression. Known cancer-associated genes in humans and mouse model systems are largely associated with cell fate, genome instability, genome maintenance, and response to stress (101). Besides any impacts from lifestyle differences, we reason that higher cancer rate in humans than in chimpanzees may be partly linked to genetic and structural variant differences that impinge on chromosomal diversification, cell-cycle progression, and lysosomal signaling (43, 102, 103, 104, 105) plus to critical differences in gene expression regulation and chromatin organization that impact responses to stress (106, 107). Besides fine-tuning gene expression patterns, it will be worthwhile to investigate whether these genomic changes, which have arisen during remarkably short evolutionary times, may have come at the expense of increased cancer susceptibility.

With GSEA enrichment factors <0.1 in healthy samples, the immunoglobulin complex (IC) and OR were the gene families with the lowest representation of pG4 motifs (Fig. 1*C*; Table S2). In contrast, neurogenesis- and brain development-related genes were at the opposite end of the GSEA spectrum with the richest representation of pG4 motifs (>1.2). As these results were based on the average expression across thousands of samples and on large lists of genes, we believe they are biologically relevant. In humans, both IC and OR genes are understood as among the most variable families of genes, both in terms of structural polymorphism and germline variation—the IC genes in response to the generation of stochastic antibody diversity, the OR genes as a likely consequence of reduced reliance on olfaction for survival (108, 109, 110, 111).

A higher rate of sequence changes has occurred in cortical development neurogenesis genes along the human lineage compared to rodents, which also led to the accelerated acquisition of new genes in great apes (112). However, most genetic changes that occurred to support cortical expansion and brain size during primate evolution appear to have targeted (1) gene regulatory elements (*i.e.* human accelerated regions (HARs), microRNAs, and long noncoding RNAs (38, 75, 113, 114), and (2) more complex topologically associated domains formed by chromatin-looping (113). Indeed, an analysis of Neanderthal and Denisovan hominin genomes suggests that the rate of changes in HARs coming to fixation in modern humans has exceeded genome-wide average (113). These changes potentially led to a concerted expression of genes physically distant in linear space (112). In comparison, we reason that the gradient in pG4-containing genes observed in our GSEA may reflect evolutionary patterns pointing toward a higher and more coordinated expression of genes related to brain, and possibly other tissues, along the human lineage, which then manifests in reduced expression variability at the population level (indiv-CV). pG4 motifs may have contributed to enhancing gene expression by acting from gene regulatory regions, including topologically associated domains (38) and, potentially, HARs. Therefore, it will be of interest to test whether the modest magnitude increase in pG4 motifs in genes related to neurogenesis and brain development has played a role in human-specific traits.

The low variation in expression of pG4-containing genes compared to genes without pG4 motifs was observed at the population level for both normal tissues and tumor samples. Notably, this relationship held even though gene expression was overall stronger in tumors than in matched controls. Tissue-specific gene expression variability has been linked to the number of transcripts, CG ratio at promoters, regions of DNase hypersensitive sites (*i.e.* accessible chromatin), and H3K4me3-marked chromatin of which all correlate with low variability (115). As mentioned, native G4 structures identified by G4-CUT&Tag coincide with these factors, and thus the low indiv-CV of the pG4-containing transcriptome is in line with these prior findings. We also observed that, for five cancers, pG4 g-CV in tumors was lower than in matched normal tissues, a pattern absent for genes without pG4, suggesting that the association between the kinetics of G4 formation and transcription (74, 75) observed in cell culture may also apply to the reprogramming of gene expression in cancer cells. The extent to which frequently occurring somatic base variants at pG4s in cancer may impinge on this reprogramming of gene expression remains to be established and should be addressed in future studies.

We find that germline mutations tend not to target pG4 motifs related to brain-specific gene functions or stable G4-forming motifs. The low mutability at stable pG4s, which occur frequently within gene bodies, is in line with the finding that active chromatin domains are generally subject to stronger repair of oxidative damage than heterochromatic regions (111). However, different sequence composition of bases within pG4 loops likely plays a role in the overall mutational rates, as shown for the simplest 1-base loop pG4 subsets (116). By contrast, somatic variants in cancer occur preferentially at stable G4-forming sequences, supporting the view from other studies for a G4 role in generating cancer structural variants (27, 117, 118). The mechanisms underlying differential pG4 mutability in germline and cancer remains an open question to be tested. Based on current data, the oxidative environment of cancer cells may contribute to this phenomenon, given the low ionization potential of G-runs (116, 119, 120, 121). The DNA sequence context in which pG4 motifs are embedded may also synergize pG4 mutagenesis, as shown by the strongest hotspots in germline and cancer, which are in a DNA context that favors local folding into metastable hairpin-loops, including the pG4 motifs themselves. Thus, such factors may lead to rare variant alleles in the germline, which then segregate through Mendelian inheritance, and to accelerated mutagenesis in the highly oxidative tumor environment (122, 123).

Alterations in gene expression programs are tightly connected to cell fate decisions. Our collective findings raise the prospect that pG4 sequence positions may contribute to both increased and regulated gene expression. Notably, as G4 is viewed as a molecular target for induced synthetic lethality in cancer cells, G4 stabilizers are being pursued in emerging clinical trials seeking novel therapeutic strategies for synthetic lethality in cancer (124). So, generally advancing knowledge of pG4 interaction pathways during transcription and replication may be valuable to weigh risk and benefit for such cancer therapies. Moreover, besides their presence in mammalian cells, G4s are predicted in most human viruses and experimentally shown in all the main virus groups, so G4 ligands have been developed and are under consideration for both cancer cells and viral pathogens to date (82). Indeed, both in cancer cells and in pathogens the combination of fast mutation rates, which makes therapeutic resistance a major challenge, and the insufficiency of effective drugs makes potentially general drug targets highly sought. Although G4 stabilizing ligands typically lack canonical “drug-like” properties, they may show accumulation and efficacy, such as seen in tumor xenografts of human cancers. These observations provide a rationale for further G4 stabilizer development for both cancer and pathogens (83), and thus for a greater understanding of G4 roles in normal and cancer cells.

Overall, our genome-wide bioinformatic analyses implicate human pG4 in robust well-coordinated transcription and reduced cancer transcriptome variation that is likely more important in humans than in mouse models and in chimpanzee. Intriguingly, our findings furthermore uncover implications of G4 in gene expression dynamics not only for model organism use but also for human biology and medicine. These computational findings may guide experimental studies to test connections between curated mutations and G4 dynamics, plus the potential role for G4 genome rewiring in driving the divergence in gene-expression and cancer risk between species.

### Limitations of the study

These analyses and bioinformatic approaches can find statistically significant associations between variables, irrespective of whether they are functionally interdependent or not. Here, we harnessed the speed of the Texas Advanced Computing Center and the very large size of the biologically derived data being queried to detect general relationships often hidden in most experimental settings. Yet, the computational results lack the means to test physical and architectural interactions that may underlie G4 relationships with gene expression. We were also unable to test mouse and chimpanzee tissue-specific associations with pG4 expression patterns due to the lack of robust expression atlases for these animal models. However, our current bioinformatic results establishing pG4 genomic associations and their implications may help focus ongoing experimental efforts across laboratories worldwide. Test experiments could for example leverage CRISPR-Cas9 technology to create informative constructs in a reasonably high-throughput way. Such experimental tests will be important to determine if the associations of human pG4 at TSS with robust coordinated transcription and reduced cancer transcriptome variation are functionally interdependent.

## Experimental procedures

### G4 DNA search algorithms

We used an in-house pure pattern-searching C++ algorithm run in a parallel environment, named C++Quad, to search for pG4 patterns in genomes extracted from NCBI (https://www.ncbi.nlm.nih.gov/) and the University of California Santa Cruz (UCSC) Genome Browser (https://genome.ucsc.edu/). The pG4 search pattern used was [G_3_N_1-7_]_>3_G_3_ (*i.e.* at least four sets of 3 G’s in a row, each separated by 1–7 bp [G4s with longer loops (>7 bps) were excluded due to predicted low stability (8)]. We implemented modifications to this search pattern depending on specific pG4s of interest. We performed the search for the human genome hg38 assembly, as well as for chimpanzee (panTro6) and mouse (mm10). We extracted EST coordinates from the UCSC Genome Browser all_est.txt files (https://hgdownload.soe.ucsc.edu/). We performed Fisher tests using an in-house script to assess whether pG4s were enriched within EST start sites.

### Gene expression analyses

We created the lists of genes containing pG4s at specific regulatory locations [*e.g.* TSS, TSS-45 (*i.e.* 45 bp before a TSS (111)], 5′UTR, 3′UTR, exons, introns) by intersecting the pG4 coordinates with the gene coordinates from UCSC Genome Browser refFlat.txt or refGene.txt files (https://hgdownload.soe.ucsc.edu/goldenPath/hg38/database/). We extracted exon coordinates from Ensembl BioMart (http://useast.ensembl.org/info/data/biomart/index.html) and used Bedtools (125) v. 2.26.0 (*intersect* command) for the intersections. We retrieved normalized gene expression data from The Human Protein Atlas (47) or TCGA (https://portal.gdc.cancer.gov/), whereby only cancer types with at least 10 matched controls were included. We used the R (https://www.r-project.org/) utilities ggplot2 v. 4.0.3, ggpubr v. 0.4.0, and pheatmap v. 1.0.12 to generate boxplots, heatmaps, and scatterplots of gene expression, and to perform statistical analyses.

### PANTHER Gene Set Enrichment Analysis

We submitted the gene lists used for gene expression analyses to the PANTHER online GO tool (http://geneontology.org/) for GSEA, and used the Bonferroni correction to assess statistical significance for the analysis of gene ontology term classifications comprising Biological Process, Molecular Function, and Cellular Component. We calculated the log2 of the fold enrichments and –log10 of the *p*-values and plotted the data using GraphPad Prism v. 8.0.0 (https://www.graphpad.com).

### Pathogenic mutations screening

We retrieved the hg38 genomic coordinates of human mutations from HGMD (126) (http://www.hgmd.cf.ac.uk/ac/index.php, June 2019 version, hgmd-hg38.bed file), and COSMIC (127) (https://cancer.sanger.ac.uk/cosmic, CosmicMutantExport_v92.tsv and Cosmic_Breakpoints_v99_GRCh38.tsv files). We used the HGMD dataset to extract confirmed disease-related germline mutations, whereas we used the COSMIC dataset to extract the genomic coordinates of “potentially cancer-causing” (pathogenic according to the Functional Analysis through Hidden Markov Models (FATHMM) prediction) as well as “potentially benign” mutations (neutral or non-evaluated according to Functional Analysis through Hidden Markov Models prediction [FATHMM]). We also extracted chromosomal breakpoints for additional analyses. We performed Fisher tests to test whether mutations were enriched within pG4s or not. We used Quadron (https://quadron.atgcdynamics.org) (128) to assess the stability scores of pG4s by setting the threshold for stable G4 structures to ≥19.

### Genome-wide prediction of metastable hairpin-loop folding structures

We divided the reference human genome sequence (hg38) into 500-base bins and on each of the bins for which no gaps were found (∼5.2 million total) we determined the ΔG of the most stable harpin/loop folding configuration using mfold (http://www.unafold.org/mfold/software/download-mfold.php) (61). We then obtained a genome-wide histogram of the ΔG distribution by plotting the number of bins found at each 5-units increments of ΔG values from −272.5 to 7.5 kcal/mol.

### Predicted hairpin-loop folding by selected pG4 sequences and mutation mapping

To assess the propensity of target pG4 sequences to fold into metastable hairpin-loop structures, we extended the pG4 sequences by 20 bp at both the 5′ and 3′ ends, analyzed the extended sequences with the online tool RNAFold (http://rna.tbi.univie.ac.at/cgi-bin/RNAWebSuite/RNAfold.cgi), and recorded the free energy of the thermodynamic ensemble. We then mapped the germline mutations from the HGMD (HGMD_Advanced_Micro_Lesions.csv and HGMD_Advanced_Substitutions.csv files) for the *MEN1* gene, or simple (single base substitutions and short insertion and deletions) somatic mutations from file CosmicMutantExport_v92.tsv for the *NOTCH1* and *PTCH1* genes, onto the folded DNA configurations. We also recorded and plotted the ΔG value for the 500-base bin (see above) containing such pG4s. To find the genomic sites with the highest numbers of simple mutations, we queried the CosmicMutantExport_v92.tsv file, selected the entries with short insertions/deletions/duplications and ranked the results using custom bash scripts.

## Data availability

C++ Quad source code is available on GitHub: https://github.com/abacolla/nonB-DNA. Pipelines used for location-specific genome-wide pG4 search and expression analysis are available on GitHub: https://doi.org/10.5281/zenodo.10557790.

## Supporting information

This article contains supporting information.

## Conflict of interest

The authors declare that they have no conflicts of interest with the contents of this article.
