# Supplementary material for: Enriched G4 forming repeats in the human genome are associated with robust well-coordinated transcription and reduced cancer transcriptome variation
Source: J Biol Chem. 2024 Sep 26;300(11):107822. doi: 10.1016/j.jbc.2024.107822 (PMC11532954; doi:10.1016/j.jbc.2024.107822)
Supplement: Supporting information [file mmc1.docx]

**SUPPORTING INFORMATION**

**Enriched G4-forming repeats in the human genome are associated with robust well-coordinated transcription and reduced cancer transcriptome variation**

Ruth B. De-Paula^1,2^, Albino Bacolla^2^, Aleem Syed^2,3^ and John A. Tainer^2,4,^*

*To whom correspondence should be addressed. Tel: +1 713 563 7725; Fax: +1 713 794 3270; Email: [jtainer@mdanderson.org](mailto:jtainer@mdanderson.org)

**Figure S1**

**
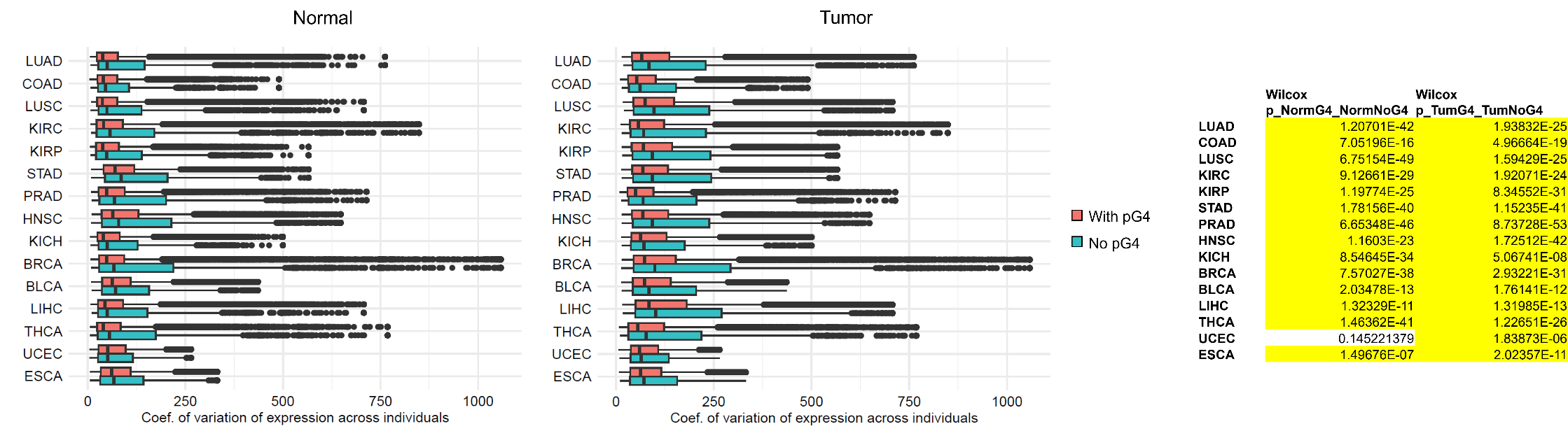
**

**
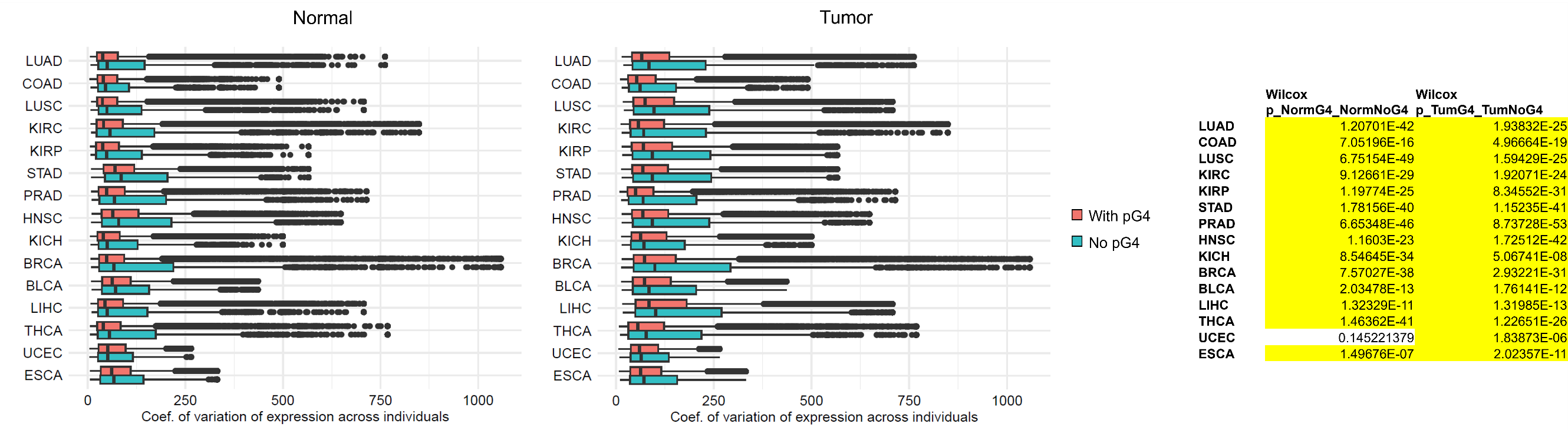
**

**Figure S1.** **Boxplots representing the comparison of the coefficient of variation of expression across individuals (indiv-CV) between genes with and without pG4.** Boxplots for genes with and without pG4s in normal (top left) and tumor (top right) tissue. Most comparisons between genes with and without pG4 were statistically significant according to Wilcoxon tests (table at the bottom). LIHC: Liver Hepatocellular Carcinoma; LUAD: Lung Adenocarcinoma; LUSC: Lung Squamous Cell Carcinoma; UCEC: Uterine Corpus Endometrial Carcinoma; KIRC: Kidney Renal Clear Cell Carcinoma; ESCA: Esophageal Carcinoma; BLCA: Bladder Urothelial Carcinoma; BRCA: Breast Carcinoma; KIRP: Kidney Renal Papillary Cell Carcinoma; THCA: Thyroid Carcinoma; STAD: Stomach Adenocarcinoma; COAD: Colon Adenocarcinoma; HNSC: Head and Neck Squamous Cell Carcinoma; PRAD: Prostate Adenocarcinoma; KICH: Kidney Chromophobe Carcinoma.

**Figure S2**

**
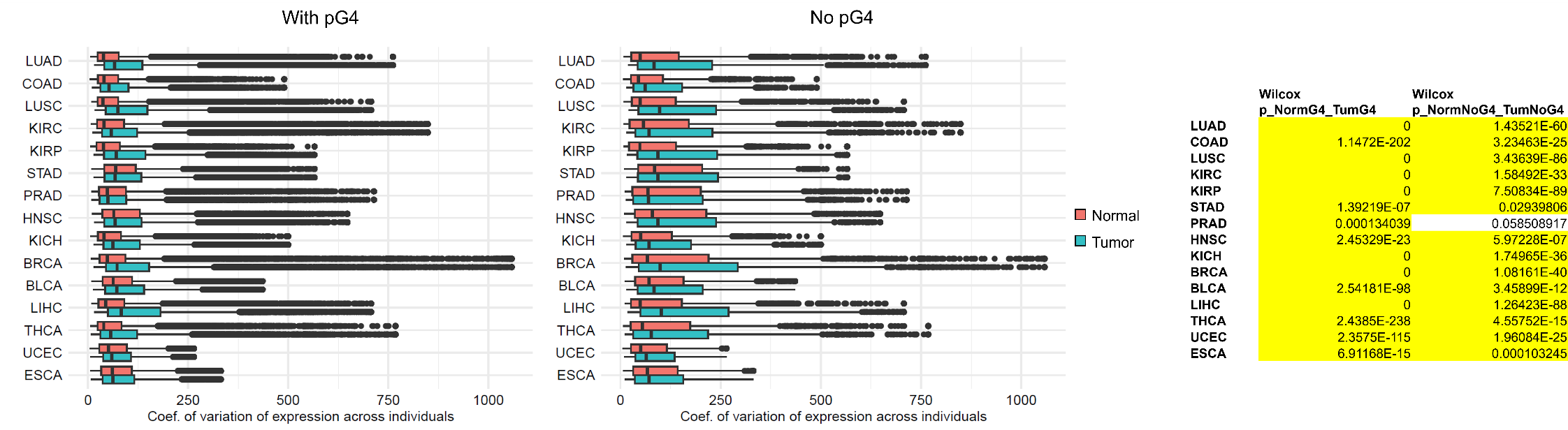
**

**
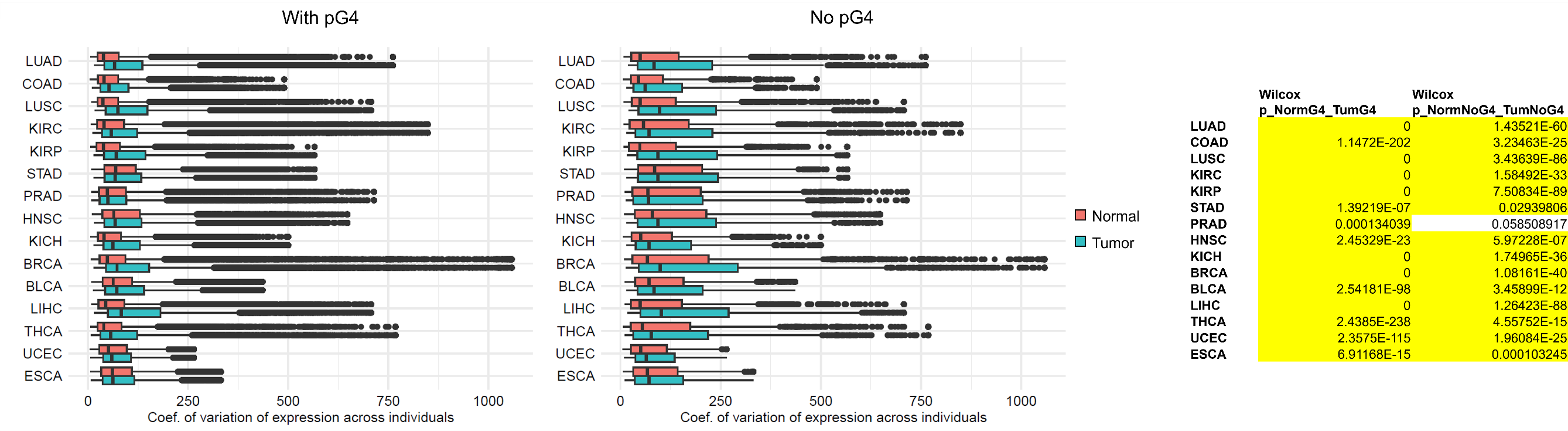
**

**Figure S2.** **Boxplots representing the comparison of the coefficient of variation of expression across individuals (indiv-CV) between normal and tumor samples.** Boxplots for normal and tumor tissues in genes with (top left) and without (top right) pG4. Most comparisons between normal and tumor tissues were statistically significant according to Wilcoxon tests (table at the bottom). LIHC: Liver Hepatocellular Carcinoma; LUAD: Lung Adenocarcinoma; LUSC: Lung Squamous Cell Carcinoma; UCEC: Uterine Corpus Endometrial Carcinoma; KIRC: Kidney Renal Clear Cell Carcinoma; ESCA: Esophageal Carcinoma; BLCA: Bladder Urothelial Carcinoma; BRCA: Breast Carcinoma; KIRP: Kidney Renal Papillary Cell Carcinoma; THCA: Thyroid Carcinoma; STAD: Stomach Adenocarcinoma; COAD: Colon Adenocarcinoma; HNSC: Head and Neck Squamous Cell Carcinoma; PRAD: Prostate Adenocarcinoma; KICH: Kidney Chromophobe Carcinoma.

**Table S1 (.xlsx): Statistical tests for The Human Protein Atlas, TCGA, HGMD and COSMIC.**

**Table S2 (.xlsx): PANTHER GSEA results for The Human Protein Atlas.**

**Table S3 (.xlsx): HGMD and COSMIC mutations within pG4.**
